# Supplementary material for: An exploratory study on excess weight gain: Experiences of Postmenopausal Women in Ghana
Source: PLoS One. 2023 Jan 13;18(1):e0278935. doi: 10.1371/journal.pone.0278935 (PMC9838829; doi:10.1371/journal.pone.0278935)
Supplement: S2 File — (DOCX) [file pone.0278935.s002.docx]

**CODES**

Don’t want to reduce. Unable to sleep. Good-looking. Feel numbness.

Beautiful. Full of medications. Proud of myself. Feel uncomfortable. Experiences health problems. Unhappy. Confidence. Sicknesses. Feel heaviness. Lazy. Always weak. Painful knee joint. Experiences sever waist pain. Nice-looking. Inactive. Full diseases. Body pains. Exhausted. Attractive. Natural body. Low self-esteem. Feel appreciated. Uncomfortable. Thumb stiffness. Extreme body pain. Experiences waist pain. Hypertension. I’m motivated. Experience palpitation. Stress-up. Always panting. Feel Normal. Painful ankle. Comfortable. Happy. Don’t care. Full of sicknesses. Admiration. Praises. Experiences migraines. feel proud. Full of huge hospital bills. Feel presentable. Experience joint pains. Anxious. Unappealing. Full of confidence. Unstable. Always tired. Appealing. Pain around my thigh. Pain killers. Profit decline. Difficult walking. Joint pains. Feel restless. Feel good. Experience high cholesterol. Feel Peace. Health challenges. Cannot walk distance. Tired easily. Difficult to get up. Satisfied. A lot of sickness. Dress well. look appealing. Good self-esteem.

**CODE CATEGORIZATION**

Feel Joint pains. Experiences chronic diseases. Feel attractive. Presentable. Feel body pains

**Code categorization**

Eat vegetables and fruits. Adjust eating habit.

Physical activity. Reduction in food quantity. Reduced starchy food and red meat. Exercise. Weight reduction remedies.

**CODES**

Walking. Exercise. TV exercise. Reduce food quantity. Reduced fufu, banku and yam. Reduce meat. Eat oranges, watermelon, mangoes. Time of eating. Warm water and lemon mixture. Drink warm water. Control the quantity of food. Eat early. Physical activities. Chores. Eat carrot, cucumber, cabbage. Slim tea. Forever product. Herbal tea. Shake. Skip breakfast. Eat dry fish. Morning walking. Drink lemon and honey. Reduce fatty food. Reduced red meat and salt. Walk and reduced fufu. Evening walking. Eat pineapple and banana. Eating early. Reduced fresh meat.

**CODE CATEGORIZATION**

Public health education on exercise and diet. Available exercise facilities. Less expensive facilities charges. Encouragement. Motivation. Support.

**CODES**

Health information. Gym centres. Diet education. Education on exercises and herbal products. Education on food to eat. Motivation. More gym centres. Free check-ups. Educations on food. Enough exercise facilities. Emotional support. Radio education. Community health program. Education via information centres. Available exercise centres. Government support. Recreational parks. Family motivation. Health screening and education. Free health insurance. Positive words. Exercise facilities. Information. Cheap exercise facilities. Type of exercise. Free medical examinations. Husband’s encouragement. Public health education. Constant education. Less expensive exercise centres. Incentives.
