# Supplementary material for: An exploratory study on excess weight gain: Experiences of Postmenopausal Women in Ghana
Source: PLoS One. 2023 Jan 13;18(1):e0278935. doi: 10.1371/journal.pone.0278935 (PMC9838829; doi:10.1371/journal.pone.0278935)
Supplement: Attachment — Submitted filename: RESPONSE TO REVIEWERS.docx [file pone.0278935.s003.docx]

Responses to academic editor and reviewer

Dear Editor,

Please find below the responses to the academic editor and reviewer on the manuscript title: An exploratory study on excess weight gain: Experiences of Postmenopausal Women in Ghana.

We sincerely thank the Reviewer for the truly helpful comments, which we have read through carefully.

Please find our detailed responses below for each comment.

**The academic editor**

Question 2

a. **Comment:** Please clarify the sources of funding (financial or material support) for your study. List the grants or organizations that supported your study, including funding received from your institution

**Response:** We received Faculty Research Committee Individual Grant from the University of the Witwatersrand, Johannesburg

b. **Comment:** State what role the funders took in the study. If the funders had no role in your study, please state: “The funders had no role in study design, data collection, and analysis, decision to publish, or preparation of the manuscript.”

**Response:** The funders had no role in study design, data collection, and analysis, decision to publish, or preparation of the manuscript

c. **Comment:** If any authors received a salary from any of your funders, please state which authors and which funders.

**Response:** No author received funds for the study

d. If you did not receive any funding for this study, please state: “The authors received no specific funding for this work.”

**Response:** The authors received no specific funding for this work

Question 3

a. **Comment:** In your Data Availability statement, you have not specified where the minimal data set underlying the results described in your manuscript can be found.

**Response:** This has been revised and found on Page 21

**Reviewer Comments to the Author**

Abstracts

1. **Comment**: Please clarify if “the society” stated in the introduction and result refers to the Ghanaian society rather than society as a whole.

**Response**: This refers to Ghanaian society and most African societies.

2. **Comment:** Suggest to bring forward the three main themes earlier in the result section

**Response:** This has been revised and highlighted in RED on Pages 2 and 3

Methods

1. **Comment:** Please clarify what inclusion criteria was used to define postmenopausal women in the study.

**Response:**

Women who have not had their period for 12 consecutive months and over the age of 45 who were not on any weight-loss therapy and did not have any physical or mental disabilities.

**Comment**: Page 7, moderator’s guide is commonly used for FGD rather than semi-structured interview guide.

**Response**: This has been revised and highlighted in RED on Page 7

**Comment**: Please clarify whether thematic analysis or content analysis was used. It was stated in page 7 that thematic analysis was performed whilst in abstract and page 8 (data analysis) that thematic content analysis was used.

**Response**: This has been revised as “thematic analysis” in the abstract and highlighted in RED

**Comment:** Stated on page 8 that de-briefing and member checking were performed to ensure the trustworthiness and credibility of the study. Please provide details on how these processes were conducted.

**Response:** As part of the debriefing process, participants were informed about the intentions of the study. Soon after each focus group session ends, the moderator read the excerpts of the discussion and exchanged insights from the focus group session including field notes taken throughout the discussion by the assistant moderator with the participants.

For member checking, key responses were summarized for participants to validate if they accurately reflected their true experiences.

**Commen**t: Page 8, I don’t understand the statement “The first author also surmised the responses of each focus group interview to facilitate an exact representation of participants’ views and experiences”. Please clarify how this was performed.

**Response:** The first author who is fluent in both Twi and English obtained the recording and listened to the discussion immediately after each focus group session. Inconsistent comments were probed for understanding. Field notes were reviewed and reports were prepared for each focus group session. Transcribed focus group discussions were provided to 2 randomly selected participants from each group to read through their responses for validation. The first author also shared the transcription for verification with other authors who were present at the focus group session.

**Commen**t: Please provide the moderator’s guide as a supplementary document.

**Response**: Thank you. This has been revised and highlighted in RED

Results

**Comment**: Page 9, total participants of the study was 24, if n=24, should it be 100%? Please rectify the frequency and percentage for “Christians” and “high school certificate”.

**Response**: Thank you for the comment, however, the percentage was used to get insight into the proportion of participants in the study population who are Christians and have completed high school.

**Comment**: Suggest to add participants’ anthropometric data in Table S1.

**Response**: Thank you. This has been revised and highlighted in RED

**Comment**: Page 11, sub-theme – diet-related changes. Consider to add information on participants’ previous diet in the description, e.g. they reduced the intake of starch and meat, and have now switches their diet to mainly fruits and vegetable.

**Response**: Thank you. This has been revised and highlighted in RED

**Comment**: Page 12, suggest to provide explanation on “fufu” in bracket, this will help international readers' better understand the context.

**Response**: Thank you. This has been revised as “Ghanaian indigenous starchy food”. Page 13

Discussions

**Commen**t: Page 18, stated that “Most of the times, the efforts and efficacy of these therapies do not produce successful outcomes”. Please provide evidence or references to support your claim.

**Response**: Please, reference has been provided and highlighted in RED
