# Supplementary material for: An exploratory study on excess weight gain: Experiences of Postmenopausal Women in Ghana
Source: PLoS One. 2023 Jan 13;18(1):e0278935. doi: 10.1371/journal.pone.0278935 (PMC9838829; doi:10.1371/journal.pone.0278935)
Supplement: Attachment — Submitted filename: Responses to the academic editor.docx [file pone.0278935.s004.docx]

Responses to the academic editor

Dear Editor,

Please find below the responses to the academic editor on the manuscript title: An exploratory study on excess weight gain: Experiences of Postmenopausal Women in Ghana.

We sincerely thank the academic editor for the truly helpful comments, which we have read through carefully.

Please find our detailed responses below for each comment.

Abstracts:

**Comment**: Please clarify if “the society” stated in introduction and result refers to the Ghanaian society rather than society as a whole.

**Response**: Please, this refers to the Ghanaian society

Methods

**Comment**: Please clarify what inclusion criteria was used to define postmenopausal women in the study.

**Response**: Please, the study included postmenopausal Ghanaian women over the age of 45 who were not on any weight-loss therapy and did not have any physical or mental disabilities. Found on page 7

**Comment**: Page 8, I don’t understand the statement “The first author also surmised the responses of each focus group interview to facilitate an exact representation of participants’ views and experiences”. Please clarify how this was performed.

**Response**: Please, “The first author was an observer in all session. He obtained the recordings and listened to the discussion immediately after each of the focus group session. He probed inconsistent comments for understanding. The first author reviewed field notes and prepares reports for each focus group session. After transcription, he randomly selects two participants from each group to read through their responses for validation.

**Introduction**

**Comment**: Please provide a reference citation for the statement on page 5, paragraph 3, i.e. “The public health interventions to address excess weight gain in Ghana have focused on diet-related changes and the adoption of physical activity.”

**Response**: Please, the reference has been provided. Found on page 7 “Aryeetey RN. Perceptions and experiences of overweight among women in the Ga East District, Ghana. Frontiers in nutrition. 2016 Jun 2; 3:13.”

**Comment**: Please add some detailed information on how data analysis was performed. Please include who performed the coding. Any software was used?

Response: Please, no software was used. Data analysis was manually done. Each author (IMB, MOM, BA) independently coded one domain from the four transcripts. The codes were presented to the moderator for verification. Coding tree attached

**Comment**: Please integrate participants’ demographic characteristics and anthropometric data into one single table.

**Response**: Please, this has been done.

**Comment**: Please add a brief sentence in the paragraph, e.g., the Participants demographic characteristics and anthropometric data are summarized in Table 1.

**Response:** Please, this has been revised

**Comment: Page** 12, line 2…enjoy seasonal fruits are grown locally, please remove “are”.

**Response:** Please, this has been revised

Discussions
**Comment:** Page 17, paragraph 2, “…..a finding supported in previous studies [24]”. The reference citation suggests that there was only one previous study. Please cross-check.

**Response**: Please, this has been revised.
