# Supplementary material for: An exploratory study on excess weight gain: Experiences of Postmenopausal Women in Ghana
Source: PLoS One. 2023 Jan 13;18(1):e0278935. doi: 10.1371/journal.pone.0278935 (PMC9838829; doi:10.1371/journal.pone.0278935)
Supplement: Attachment — Submitted filename: MODERATORS QUIDE.docx [file pone.0278935.s005.docx]

**The moderator’s guide**

**Introduction**

I would like to thank you for participating in this focus group discussion. The objective will be to explore excess weight gain among postmenopausal. You have been asked to participate in this focus group discussion. I expect this discussion to last for about 45 minutes to 1 hour.

Questions

1. What is your thoughts regarding your current weight?

2. Are you concern about your current weight?

If you are concerned, why

If you are not concerned, why

3. What are the things you do to manage your weight?

Probe question

a.	How often?

b.	When do you do these?

c.	How do you do these?

d.	Why?

4. What type of support would help you to adhere to a weight management	programme?
